# Veterinary Evidence: moving forward

**DOI:** 10.18849/ve.v9i3.707

**Published:** 2024-09-18

**Authors:** Peter Cockcroft

**Affiliations:** School of Veterinary Medicine, University of Surrey, United Kingdom

**Keywords:** knowledge summaries, review articles, statistics

## Abstract

Unrecognised and unknown information needs can significantly impact on the quality of decision making, advice, and patient care. In the practice of veterinary medicine, additional information may be required in one or more of epidemiology, diagnosis, treatment, prognosis, control (risk reduction), and prevention (risk avoidance), in order to optimise patient care.

*Veterinary Evidence* publishes Knowledge Summaries, which aim to answer a specific and focused clinical query by appraising the best available evidence published in peer-reviewed scientific journals. By presenting the evidence in a structured and easily digestible format, Knowledge Summaries enable veterinary professionals to stay informed and apply the best available evidence to their decision-making.

To assist in the identification of unrecognised deficits and the generation of clinical queries *Veterinary Evidence* has developed a list of common conditions for each species, alongside the different categories of information. The aim is to prompt and support the generation of new clinical queries in a more systematic format. This resource sits alongside our species-based lists of clinical queries that have already been suggested by practicing veterinarians, veterinary nurses, and other paraprofessionals (see veterinaryevidence.org/index.php/ve/clinical-queries). We hope readers and prospective authors will use these resources to generate new clinical queries or as a starting point for writing their own Knowledge Summaries.

Most of the papers published in *Veterinary Evidence* are Knowledge Summaries, but the journal has always accepted and will continue to accept and encourage other types of reviews and original research that provide evidence to inform clinical practice. For example, in this issue we have published a commentary paper by Professor Jonathan Elliott regarding the processes and evidence required for the registration of veterinary medicinal products which provides some important insights and debate into the publication of evidential information (Elliott, 2024).

A Knowledge Summary may not always be the best format to synthesise and summarise evidence on a multifaceted topic; scoping reviews, systematic reviews, or narrative reviews maybe more appropriate. Munn et al. (2018) provide a useful account of scoping reviews and how they differ from traditional literature and systematic reviews. In particular, we are keen to receive articles on artificial intelligence applications, sustainability, and quality improvement in clinical practice, as the impact and importance of these topics grows.

We are also making some changes to the presentation of Knowledge Summaries in *Veterinary Evidence*, to help readers more easily understand the details and context of the summarised evidence. In order to support everyone in appraising and understanding quantitative clinical studies, we will be publishing a glossary and guidance on basic statistical terms and tests on the journal website.

Unrecognised and unknown information needs can significantly impact on the quality of decision making, advice, and patient care. In the practice of veterinary medicine, additional information may be required in one or more of epidemiology, diagnosis, treatment, prognosis, control (risk reduction), and prevention (risk avoidance), in order to optimise patient care.

*Veterinary Evidence* publishes Knowledge Summaries, which aim to answer a specific and focused clinical query by appraising the best available evidence published in peer-reviewed scientific journals. By presenting the evidence in a structured and easily digestible format, Knowledge Summaries enable veterinary professionals to stay informed and apply the best available evidence to their decision-making.

To assist in the identification of unrecognised deficits and the generation of clinical queries *Veterinary Evidence* has developed a list of common conditions for each species, alongside the different categories of information. The aim is to prompt and support the generation of new clinical queries in a more systematic format. This resource sits alongside our species-based lists of clinical queries that have already been suggested by practicing veterinarians, veterinary nurses, and other paraprofessionals (see veterinaryevidence.org/index.php/ve/clinical-queries). We hope readers and prospective authors will use these resources to generate new clinical queries or as a starting point for writing their own Knowledge Summaries.

Most of the papers published in *Veterinary Evidence* are Knowledge Summaries, but the journal has always accepted and will continue to accept and encourage other types of reviews and original research that provide evidence to inform clinical practice. For example, in this issue we have published a commentary paper by Professor Jonathan Elliott regarding the processes and evidence required for the registration of veterinary medicinal products which provides some important insights and debate into the publication of evidential information (Elliott, 2024).

A Knowledge Summary may not always be the best format to synthesise and summarise evidence on a multifaceted topic; scoping reviews, systematic reviews, or narrative reviews maybe more appropriate. Munn et al. (2018) provide a useful account of scoping reviews and how they differ from traditional literature and systematic reviews. In particular, we are keen to receive articles on artificial intelligence applications, sustainability, and quality improvement in clinical practice, as the impact and importance of these topics grows.

We are also making some changes to the presentation of Knowledge Summaries in *Veterinary Evidence*, to help readers more easily understand the details and context of the summarised evidence. In order to support everyone in appraising and understanding quantitative clinical studies, we will be publishing a glossary and guidance on basic statistical terms and tests on the journal website.

## ORCiD

Peter Cockcroft: https://orcid.org/0000-0003-0090-07060

## Disclaimer

Knowledge Summaries are a peer-reviewed article type which aims to answer a clinical question based on the best available current evidence. It does not override the responsibility of the practitioner. Informed decisions should be made by considering such factors as individual clinical expertise and judgement along with patient’s circumstances and owners’ values. Knowledge Summaries are a resource to help inform and any opinions expressed within the Knowledge Summaries are the author’s own and do not necessarily reflect the view of the RCVS Knowledge. Authors are responsible for the accuracy of the content. While the Editor and Publisher believe that all content herein are in accord with current recommendations and practice at the time of publication, they accept no legal responsibility for any errors or omissions, and make no warranty, express or implied, with respect to material contained within. For further information please refer to our Terms of Use.

## References

[BIBR-1] Elliott J. (2024). Publication of registration studies in peer-reviewed academic journals. Veterinary Evidence.

[BIBR-2] Munn Z., Peters M.D.J., Stern C., Tufanaru C., McArthur A., Aromataris E. (2018). Systematic review or scoping review? Guidance for authors when choosing between a systematic or scoping review approach. BMC Medical Research Methodology.

